# Archetype-based conversion of EHR content models: pilot experience with a regional EHR system

**DOI:** 10.1186/1472-6947-9-33

**Published:** 2009-07-01

**Authors:** Rong Chen, Gunnar O Klein, Erik Sundvall, Daniel Karlsson, Hans Åhlfeldt

**Affiliations:** 1 Department of Biomedical Engineering, Linköping University, Linköping, Sweden; 2 Cambio Healthcare System, Linköping, Sweden; 3 Department of Microbiology, Tumor and Cell Biology, Karolinska Institutet, Solna, Sweden

## Abstract

**Background:**

Exchange of Electronic Health Record (EHR) data between systems from different suppliers is a major challenge. EHR communication based on archetype methodology has been developed by openEHR and CEN/ISO. The experience of using archetypes in deployed EHR systems is quite limited today. Currently deployed EHR systems with large user bases have their own proprietary way of representing clinical content using various models. This study was designed to investigate the feasibility of representing EHR content models from a regional EHR system as openEHR archetypes and inversely to convert archetypes to the proprietary format.

**Methods:**

The openEHR EHR Reference Model (RM) and Archetype Model (AM) specifications were used. The template model of the Cambio COSMIC, a regional EHR product from Sweden, was analyzed and compared to the openEHR RM and AM. This study was focused on the convertibility of the EHR semantic models. A semantic mapping between the openEHR RM/AM and the COSMIC template model was produced and used as the basis for developing prototype software that performs automated bi-directional conversion between openEHR archetypes and COSMIC templates.

**Results:**

Automated bi-directional conversion between openEHR archetype format and COSMIC template format has been achieved. Several archetypes from the openEHR Clinical Knowledge Repository have been imported into COSMIC, preserving most of the structural and terminology related constraints. COSMIC templates from a large regional installation were successfully converted into the openEHR archetype format. The conversion from the COSMIC templates into archetype format preserves nearly all structural and semantic definitions of the original content models. A strategy of gradually adding archetype support to legacy EHR systems was formulated in order to allow sharing of clinical content models defined using different formats.

**Conclusion:**

The openEHR RM and AM are expressive enough to represent the existing clinical content models from the template based EHR system tested and legacy content models can automatically be converted to archetype format for sharing of knowledge. With some limitations, internationally available archetypes could be converted to the legacy EHR models. Archetype support can be added to legacy EHR systems in an incremental way allowing a migration path to interoperability based on standards.

## Background

Exchange of Electronic Health Record (EHR) data between systems from different suppliers is a major requirement and challenge for distributed health care computing. The two-level modelling paradigm using a standard reference model and archetypes [1] defining specific clinical content models has been developed by the openEHR foundation [2] and standardized by CEN and ISO in the EN/ISO 13606 standard series [3]. This approach to standards and interoperability recognizes that there is a need and possibility for general agreement on the basic structure and structural elements of an EHR (the Reference Model). However, it also acknowledges that there is a need for various clinical expert groups to agree on specific data sets for different purposes which must be easy to amend as new requirements emerge. This can be achieved using the archetype structures which act as description of a building block of instantiated record information. So far only limited experience has been reported on exchange of clinical information between different systems that are based on the new paradigm [4].

In Sweden, the EN 13606 standard has been selected for the national projects and specifically for the National Patient Overview project [5] to exchange a summary of EHR data. However, so far none of the existing EHR products used in primary care or in hospitals have been built on the archetype methodology. Other national projects are also working on a set of standardized archetypes to define data sets to report to national quality registries for a number of health issues.

There are also other strategic plans to support exchange of clinical information between different care provider organisations for shared care facilitated by the new Swedish Patient Data Act that became effective as of July 2008. The Patient Data Act puts new pressure on EHR system vendors to support open standards, such as the EN 13606, the standard chosen by the Swedish Association of Local Authorities and Regions for EHR communication [6]. As a necessary step to achieve semantic interoperability using this standard, the semantic definition of the EHR data in the form of archetypes needs to be made available for sharing and interpretation. While the new and partly international work on designing new archetypes for various purposes has started, great value exists in the wealth of clinical models in current EHR systems, by which years of patient data have been recorded. Not only do these represent a lot of clinical information, in this case from several regional installations in Sweden, but also there is a great value of the existing consensus work by regional or national clinicians agreeing on the most important data to collect and how to structure them. Such clinical content models will be valuable inputs to development of national and international level libraries of archetypes intended to facilitate data sharing and reuse.

The openEHR RM may largely be considered a super-set of the RM of EN/ISO 13606 and the openEHR AM is identical to that of EN/ISO 13606. Some differences will have to considered when mapping the two specifications, but it is generally accepted that the two standards are compatible [7]. Due to more available software tools and clinical archetypes of openEHR format than those of EN/ISO 13606, the conversion study was performed on openEHR archetypes instead of EN/ISO 13606 archetypes.

Developed by openEHR members, the openEHR Template Model (TM) [8] has been proposed as a way of grouping and customizing archetypes tailored for local clinical settings. The TM is currently on the roadmap of the openEHR design specification project but is not yet released as version 1.0. Essentially, the openEHR TM is an extension of the openEHR AM, therefore openEHR templates technically work almost like archetypes. Due to the draft development status of the openEHR TM specification and the lack of software implementation, openEHR templates were not used in this study.

### Objectives

The objective of this study was to investigate the feasibility of transforming EHR templates from an installed Cambio COSMIC system http://www.cambiosys.com/, a regional EHR product present in Sweden and several other countries, into openEHR archetypes and inversely the feasibility of converting standard archetypes into the COSMIC template formalism.

## Methods

The template model of the Cambio COSMIC Medical Record application was analyzed and compared to the openEHR Reference Model [9] and Archetype Model [10]. A semantic mapping was developed to enable the bi-directional conversion between COSMIC templates and openEHR archetypes. Prototype software was developed based on the conversion rules and integrated in a production version of the COSMIC software to enable automated bi-directional conversion between COSMIC templates and openEHR Archetypes. The design specifications from openEHR Release 1.0.1, were used. Open source software components from the openEHR Reference Java Implementation Project [11] were used to facilitate the software development. Archetype Editors from Ocean Informatics https://wiki.oceaninformatics.com/confluence/display/TTL/Archetype+Editor+Releases and Linköping University http://www.imt.liu.se/mi/ehr/tools/ were used to view and compare generated archetypes to COSMIC templates.

Fifteen archetypes (Table 1) from the openEHR Knowledge Repository were used in this study and converted to the COSMIC template format. They were selected in the context of the Danish national archetype trial project [12] and were representative of the archetypes, in terms of constrained RM classes and clinical settings, from the openEHR archetype repository. Some of them were included due to dynamic inclusion mechanism in the archetypes.

**Table 1:** Selected Archetypes for Import In this Study

**No**.	ArchetypeID
**1**	openEHR-EHR-ACTION.imaging.v1.adl
**2**	openEHR-EHR-CLUSTER.exam-fetus.v1.adl
**3**	openEHR-EHR-CLUSTER.exam-uterus.v1.adl
**4**	openEHR-EHR-EVALUATION.problem-diagnosis.v1.adl
**5**	openEHR-EHR-INSTRUCTION.imaging.v1.adl
**6**	openEHR-EHR-OBSERVATION.body_temperature.v1.adl
**7**	openEHR-EHR-OBSERVATION.imaging.v1.adl
**8**	openEHR-EHR-CLUSTER.auscultation.v1.adl
**9**	openEHR-EHR-CLUSTER.dimensions.v1.adl
**10**	openEHR-EHR-CLUSTER.exam-generic.v1.adl
**11**	openEHR-EHR-CLUSTER.exam.v1.adl
**12**	openEHR-EHR-CLUSTER.palpation.v1.adl
**13**	openEHR-EHR-CLUSTER.percussion.v1.adl
**14**	openEHR-EHR-CLUSTER.size.v1.adl
**15**	openEHR-EHR-ITEM_TREE.imaging.v1.adl

Eighty-six COSMIC templates from the Swedish County of Östergötland, a Cambio regional EHR customer, were used as the input for the conversion to archetypes. These templates were used for clinical documentation both for primary and specialised care in the region.

### The COSMIC Template Model

**Figure 1:**
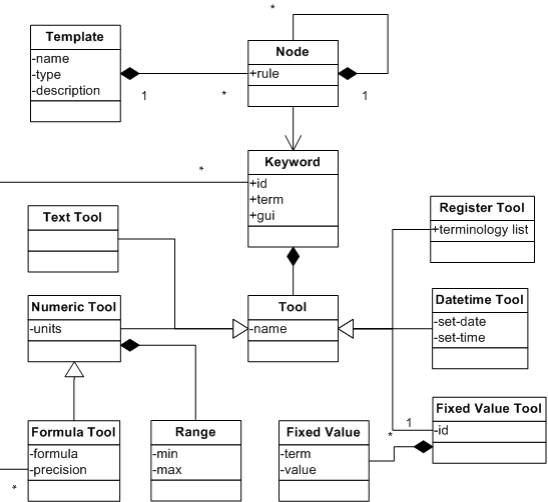
**The Class Diagram of COSMIC Template Model**.

The Cambio COSMIC template model is designed to represent clinical requirements for data entry, retrieval and display for various clinical settings, e.g. an inpatient admission form and a progress note. The COSMIC template model provides necessary structures for logically grouping different components for clinical recording and validation rules for data entries. Templates can be included inside other templates to encourage reuse. Terminology bindings between reference terminologies and data entry nodes are also supported. Data entry nodes, known also as *keywords *in COSMIC, are organized as a hierarchical structure in a template. Each data entry node can be assigned a data type with user-definable validation rules. The details of the template model design are presented in Figure 1. Note that in order not to burden the readers with unnecessary details, e.g. related to specific COSMIC application logic or user interface rendering hints, the COSMIC template model presented in this study was a subset compared to that of the runtime system. However, all model designs related to representation of template semantics, which is the focus of this study, remained intact. Keywords are reusable between different templates. Terms from locally defined terminologies or external reference terminologies can be bound to different entry data nodes or a list of pre-defined coded values.

### The openEHR Reference Model and Archetype Model

Unlike the COSMIC templates, the openEHR archetypes assume an underlying explicit Reference Model (RM) whereas in COSMIC templates, the notion of the underlying information model to process the template constraints is implicit. The openEHR RM provides support for different data types, data structures and common design choices for EHR representations. The Archetype Model (AM) provides ways of expressing constraints on the underlying RM by choosing specific RM classes, attributes and value ranges. The combined RM and AM provide the support for clinical content modelling with great flexibility and expressiveness.

### Conversion Validation Protocol

To verify the conversion result, a validation protocol was defined to manually check the spatial structure, data value constraints and terminology related constraints of the archetype representations using the Archetype Editor compared with the COSMIC templates in their native authoring environment.

The following items are checked in the validation process of each conversion:

1. Identification of COSMIC templates and archetypes.

2. Structural constraints in terms of parent-child node relationship and sibling node relationship.

3. Data value constraints in terms of correct data types of data entry nodes and associated validation rules.

4. Terminology related constraints: term definitions, terminology binding constraints and correct identification of source terminology and individual codes/terms.

## Results

### Identification and Meta-data

The identification of the openEHR archetypes and COSMIC templates is achieved by ARCHETYPE_IDs and unique template names respectively. The conversion between the two was possible based on a simple scheme. Due to practical reasons (discussed below), the openEHR RM class Evaluation was always used as part of the resulting ARCHETYPE_ID from a COSMIC template name. Each archetype has an integral part called ARCHETYPE_DESCRIPTION that is dedicated to the meta-data of the archetype. In contrast, each COSMIC template has a text description attribute and a type attribute used for relevant application logic. Due to the limited scope of this study, the conversion rules for the meta-data were not investigated.

### Semantic Mappings

The analysis of the COSMIC template model and the openEHR RM/AM has led to the categorization of the semantic mappings necessary for the conversion between the two models into three distinctive categories:

1. Structural Constraints: the mapping for structural constraints from the two models needs to be established to obtain matching structural form of the models. The hierarchical way of organizing data entry nodes according to clinical requirements need to be preserved both for human recognition and data processing based on a path-based syntax.

2. Data Value Constraints: specific rules for data validation associated with data entry nodes need to be faithfully converted. These rules are mostly dependent on the underlying data types and expressiveness of the data constraint mechanism, and are vital for achieving data quality.

3. The terminology bindings in the two models are crucial for data sharing between systems and secondary use of the EHR data based on aggregation, e.g. for epidemiology purposes. Although still not common in practise, reasoning based on reference terminologies and ontologies is expected to become one of the major benefits of terminology bindings of the clinical content models.

### EHR Entry Type for COSMIC Templates

In the RM of openEHR and EN/ISO 13606, a clinical statement is recorded in an Entry with its full context so it can be safely interpreted and reused. In openEHR the ENTRY class is further specialized into OBSERVATION, EVALUATION, INSTRUCTION and ACTION sub-classes. Each has its own distinctive clinical semantics based on a clinical record ontology as explained by Heard et al. [13]. The CARE_ENTRY class, which is the root class of all specific entry types developed according to the clinical record ontology, has a list of attributes dedicated to recording of the context of the clinical statement. Most of them are optional and can be managed by the supporting classes to the COSMIC template model. For instance, the COSMIC CONTACT class records most of the context information relevant to a clinical consultation and can be linked to the resulting records using the COSMIC template. Choosing the correct openEHR CARE_ENTRY type for COSMIC templates when converting them into archetypes however, is a challenging task. Since archetypes are intended to be reused as content models both for screen forms and messages, some of them are intentionally made small but self-containing to encourage reuse across different applications. COSMIC templates are entirely authored to support on-screen clinical documentation and sometimes to mimic paper-based forms previously used in the clinical settings. Thus most existing COSMIC templates have a larger scope than most single archetypes. Moreover, some specific clinical statements, e.g. lab investigation orders and medication orders are handled by specialised applications that are not using the COSMIC template model. Handling of different state transitions during an execution of an order requires special application logic and is thus beyond the scope of mere documenting the orders. These reasons contributed to our design choice to map all COSMIC templates into openEHR RM EVALUATION class in this study. With proper manual inspection, it should be possible to use a COMPOSITION and a number of ENTRY subclasses to represent the clinical statements of different natural classes, e.g. OBSERVATION and INSTRUCTION in a COSMIC template. The automation of such conversion remains to be explored.

These considerations lead us to focus on studying semantic mapping in the following three areas: structural constraints, data value constraints and terminology constraints.

### Structural Constraints Mapping

In the COSMIC template model, a generic tree-like hierarchical structure is supported by nesting data entry nodes, the keywords. At runtime, a clinical entry form is generated based on the hierarchical structure of the template. The clinical users can spontaneously add new keywords as they see fit in the context of recording. It is possible to specify the optionality of the data entry node on the template level. In the openEHR RM and AM, the support for structures is mainly based on the underlying data structure model, which is part of the openEHR RM. The supported spatial structures are the ITEM_SINGLE, ITEM_LIST, ITEM_TREE, ITEM_TABLE openEHR RM classes. Further constraints from the AM can be expressed to specify the optionality of data nodes on the level where the node resides. Cardinality constraints are also supported in the AM to specify the semantics for container objects whose attributes are multi-valued. Besides those structural constraints, the AM also allows the specification of the number of occurrences of a specific child node within a container object. The different data structures from the openEHR RM can easily be represented with a generic tree structure. However, the exact semantics of sibling level existence, cardinality and occurrences from openEHR AM are not easy to map to the COSMIC template model.

There is a mechanism in the AM, called ARCHETYPE_INTERNAL_REF that allows reuse of a block of archetype constraint definition in different places within the same archetype. Such a reuse mechanism is not directly supported by the current COSMIC template model. In order to achieve a similar runtime behaviour based on this semantic constraint, the source constraint block can be replicated at different target locations within a COSMIC template. This strategy could work in an automated conversion process, but would be too tedious or prone to mistakes if done manually.

There is another archetype constraint that is designed to encourage reuse between archetypes. The constraint ARCHETYPE_SLOT allows one or a list of archetypes to be included within an archetype definition. Dynamic inclusion can be achieved using a regular expression based archetype identification match in the slot. This archetype inclusion mechanism is similar to the COSMIC template-in-template feature, which allows sub-templates to be included in the main template to encourage reuse of templates. But the COSMIC template inclusion mechanism does not allow a sub-template to include further sub-templates whereas the archetype slot inclusion has no such limitation. Secondly, the identification of sub-templates has to be specific in COSMIC versus the dynamic inclusion criteria of archetype slots.

### Data Value Constraints Mapping

The data value constraints are achieved by different "tool types" in the COSMIC template model. These tool types are corresponding to the assumed data types from the openEHR Data Types model while the latter are more extensive and more detailed (Table 2). Different validation rules associated with the tool types in the COSMIC template model can be compared with different constraint mechanisms in the AM. Again, the constraint mechanisms in the AM are more expressive than the ones of the COSMIC template model.

**Table 2:** Mapping of the Data Types

openEHR Data Type	COSMIC Tool Type	Comments
DV_BOOLEAN	checkbox	
DV_STATE	NONE	
DV_IDENTIFIER	Text	
DV_COUNT	Numeric	
DV_INTERVAL	NONE	
DV_PROPORTION	NONE	
DV_ORDINAL	Fixed value list	Missing ordinal value; sorted on the integer
DV_QUANTITY	Numeric	
DV_TEXT	Text	Missing regular expression support
DV_CODED_TEXT	Fixed value list	Partially through fixed values list
DV_PARAGRAPH	NONE	
DV_DATE	Date and Time	Missing ISO pattern
DV_DATETIME	Date and Time	Missing ISO pattern
DV_TIME	Date and Time	Missing ISO pattern
DV_DURATION	NONE	
DV_MULTIMEDIA	NONE	
DV_PARSABLE	NONE	

### Terminology Related Constraints Mapping

The support for terminology related constraints exists in three forms in COSMIC:

1. Each keyword can be linked to a term from a locally defined vocabulary or an externally maintained reference terminology. Since COSMIC templates are constructed with keywords in a hierarchical structure, the navigational path to reach each leaf keyword always starts with the template root keyword and all the way to the keyword where data entry occurs. This gives the data entry node a specific context of the data entry. For example, the keyword "diabetes" under the parent keyword of "family history" would have a different clinical meaning compared to the same keyword residing under the keyword "diagnosis". This way of combining generic terms for more refined use can be seen as a form of post-coordination of terms. Similar support exists in archetypes in the form of archetype nodes and associated archetype term definitions.

2. At data entry node, a keyword can be assigned to a tool type called "register tool", which is dedicated to the task of selecting relevant terms or codes from a list of specified terminologies at the point of data entry. This feature in the COSMIC template is comparable to the constraint definition mechanism in archetypes. The difference is that with archetype constraint definition, it is possible to define a query to retrieve terms from a terminology service. The syntax of such query language is not part of the openEHR specifications yet. Because of this uncertainty, currently this part of the Archetype mechanism has not been tested extensively.

3. Also at the data entry node of a COSMIC template, it is possible to use the "fixed value" tool type, which allows the user to pre-define a list of choices, each coded by a term from a local or external terminology. The choice can be single or multiple and it is also possible to define a list of terms from different source terminologies. This feature is very similar to how C_CODED_PHRASE constrains a coded text value in archetypes. The only notable difference here is that in the archetype model it is only possible to specify a single terminology with the C_CODED_PHRASE whereas in COSMIC fixed value tool type, terms from different terminology can be mixed in the pre-defined list. Still by combining several C_CODED_PHRASE constraints each for one terminology as alternatives to a single value attribute constraint, it is possible to achieve what can be done with a COSMIC fixed value tool type with multiple terminologies. In archetype formalism, besides single archetype node based term bindings, there is also a path-based term binding, which allows the use of internally reused nodes uniquely identified by a path. Such path-based identification of the nodes is not necessary in a COSMIC template if all internally reused archetype nodes are sufficiently replicated and assigned with unique identifications. In summary, all these three forms of terminology binding in COSMIC template can be supported by archetype formalism in one way or the other.

The semantic mapping between the COSMIC template model and the openEHR model shows that the latter is expressive enough to represent the semantics from COSMIC templates, both on the structural level, leaf data types and bindings between individual data entry points and external reference terminologies.

**Figure 2:**
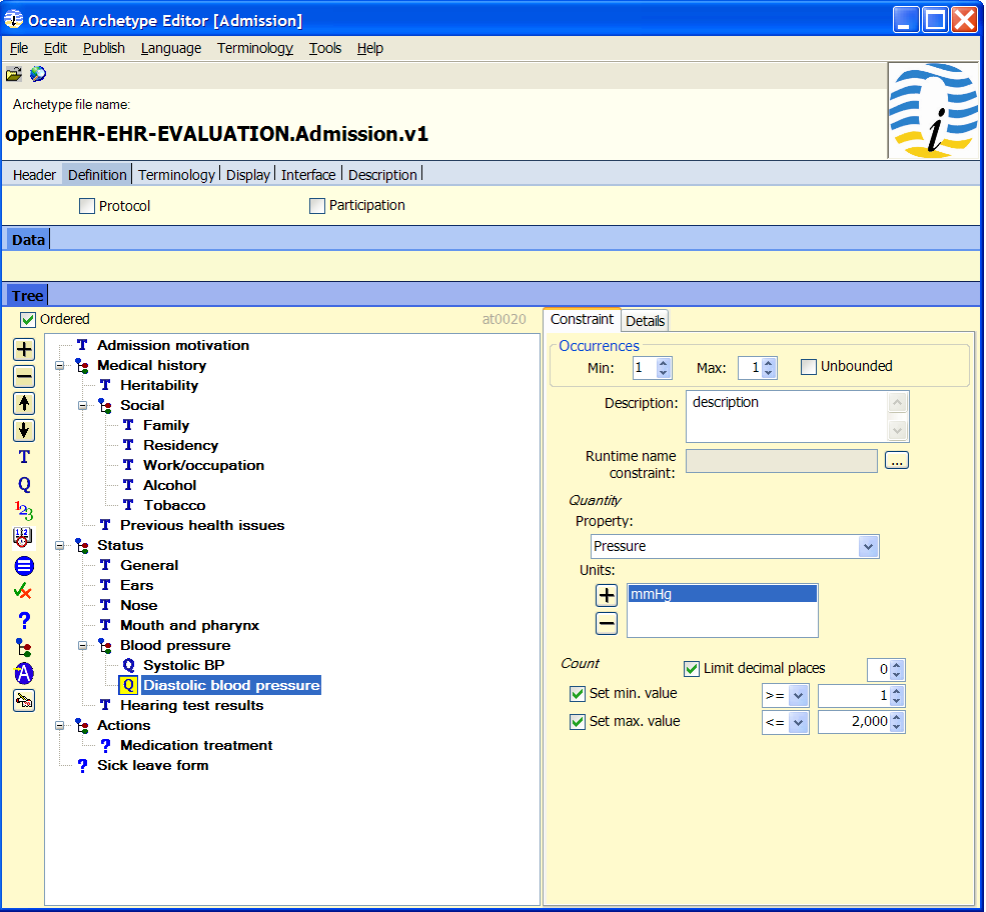
**The Exported COSMIC template in the form of an archetype**.

The prototype was developed and integrated into the COSMIC Medical Record application. COSMIC templates can be converted into archetypes compliant with the openEHR RM and AM. The resulting archetypes can be directly inspected in the two existing openEHR archetype editors (Figure 2).

### COSMIC Template to Archetype Conversion

**Figure 3:**
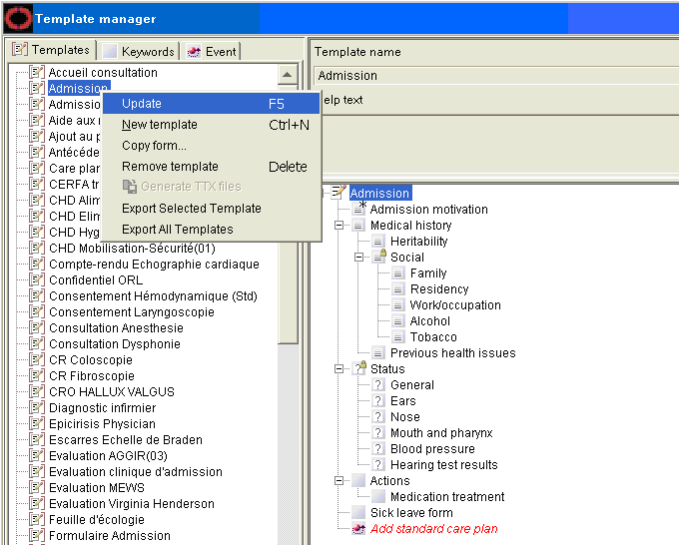
**The COSMIC Template Archetype Conversion Prototype Software**.

The prototype software for converting COSMIC templates into openEHR archetypes based on the semantic mappings presented above was integrated into the COSMIC template authoring environment (Figure 3). In the main user interface for managing templates, there is a panel that lists all existing templates in the system. By right clicking the individual template, a popup menu will appear, on which there is a choice to convert ("Export") the selected template to Archetype Definition Language (ADL) format and store the output on the file system. The conversion from a COSMIC template into an openEHR archetype is a fully automated process and does not require any manual intervention. It is also possible to launch the tool in a batch mode and convert all templates into archetypes at once.

A total number of 86 COSMIC templates were successfully converted into archetypes from the live production environment supporting the regional EHR system at the Swedish County of Östergötland. Using the validation protocol listed earlier, it was found that the original semantic definitions from the COSMIC templates were well preserved in the converted archetypes and they could be opened with an archetype editor. The visual rendering of the resulted archetypes in the Archetype Editor is similar to the rendering of the original COSMIC templates in their native authoring environment due to preservation of the spatial structure in the content model.

There are a few features of the COSMIC templates that are currently not supported by the openEHR archetype formalism. For example, it is possible to assign keywords with tool types that are dedicated to the integration with MS Word, Adobe PDF and other COSMIC application modules. These features are more related to graphic rendering or specific application logic than semantic definitions required to capture clinical requirements. Therefore these COSMIC templates features are intentionally omitted in this archetype conversion study and hence are not considered as semantic loss during the conversion of the two formats.

Nevertheless, because the COSMIC template model is less expressive in comparison to the openEHR archetype model in the areas of data value constraints, spatial structural constraints and terminology related constraints, the possibility of successful round-trip conversion depends on whether the subject of conversion falls in the shared subset of supported constraints from the two formalisms.

### Archetype to COSMIC Template Conversion

**Figure 4:**
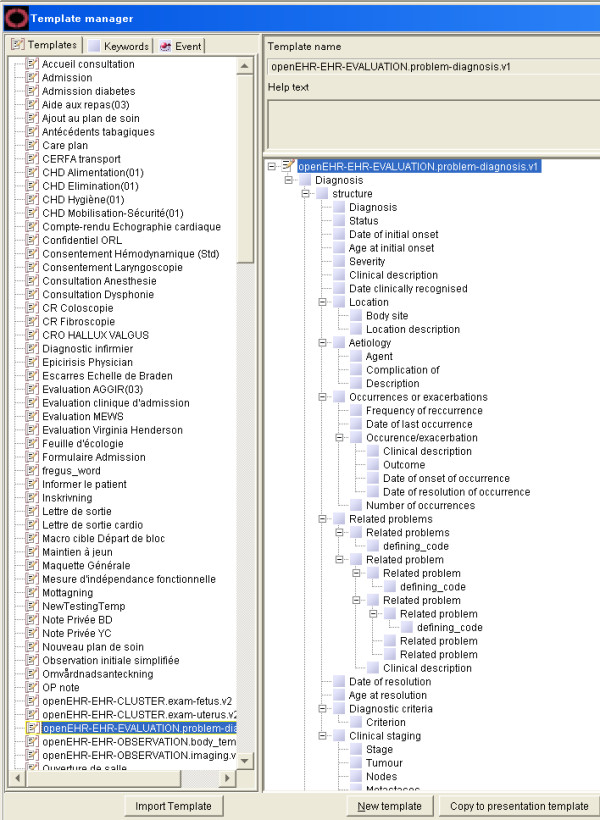
**Imported openEHR-EHR-EVALUATION.problem-diagnosis Archetype in the COSMIC Template Editor**.

**Figure 5:**
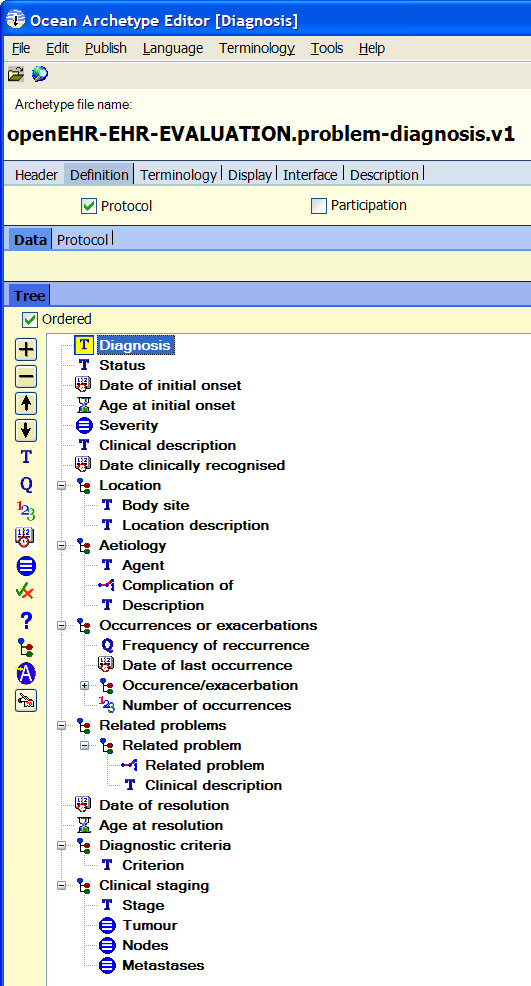
**The openEHR-EHR-EVALUATION.problem-diagnosis Archetype in the Ocean Archetype Editor**.

Prototype software (Figure 3) for conversion from openEHR archetypes to COSMIC templates based on the proposed semantic mappings was developed. During the conversion process, all necessary term definitions, COSMIC keywords and template definition could be auto-generated. The resulting COSMIC templates can be viewed directly using the native template authoring environment and used for clinical documentation. Figure 4 shows how the converted archetype openEHR-EHR-EVALUATION.problem-diagnosis.v1.adl is displayed in the COSMIC template authoring environment. The left side panel lists the name of managed COSMIC templates. The right side panel displays the hierarchical structure of the selected template, which looks very similar to how it is rendered in the Archetype Editor (Figure 5). As explained in the constraints mapping sections above, there are known openEHR archetype constraints that are currently not supported by the COSMIC template model.

Fifteen archetypes (listed in Table 1) from the openEHR knowledge repository named by the Danish archetypes trial project were successfully imported into COSMIC. The conversions were validated using the protocol listed earlier in the Methods section. Not only do these imported archetypes look similar to how they are rendered in Archetype Editors (Figure 4, 5), but also they can be used just as any other COSMIC templates for clinical documentation. Danish translation of the archetype was handled by generating a local terminology that includes all the term definitions in Danish. The names of the terms are directly used for screen rendering of the COSMIC templates and resulting clinical records. This fits very well with the intended purpose of archetype term definitions as the so called interface terminologies [14].

### A Strategy of Adding Archetype Support in Legacy EHRs

Based on the study performed on conversion of clinical models between the openEHR archetype format and the COSMIC template format, a strategy to add archetype support in current generation EHR systems was formulated. The aim is to introduce the support for archetypes incrementally through a series of manageable steps instead of a radical switch to archetype based systems disrupting the current running EHR installations. Such a practical strategy is not only useful for existing EHR suppliers but also important for regional health authorities and decision makers to allow coordinated efforts of gradually introducing standard-based and collectively developed EHR content models, e.g. using national libraries and frameworks of archetypes.

The strategy can be summarized by the following logical steps:

1. Study the bi-directional conversion of clinical content models between the archetype based format (both RM and AM) and any target EHR content format. The first step will confirm whether clinical content from the target EHR system can be safely represented using the archetype format and therefore preserving the legacy clinical content based on standards. It will also reveal the gaps, most likely in the legacy EHR model highlighting the areas that need to be improved in the target EHR system.

2. The gaps need to be carefully categorized using the criteria proposed by this study, namely data types, data structures and terminology related constraints. Once the semantic mappings are established in these three areas, a safe zone where both formats overlap will become explicit and can be used for sharing and exchange of clinical content model using archetypes with recognized limitations.

3. Gradually improvement of the target EHR model should be made in order to increase the support for the archetype format. Among the three categories of constraints, data types and data structures are probably the easiest ones to improve and will immediate give the benefit of increased expressivity. Simple forms of terminology bindings, e.g. explicit binding of one data entry to a single term could also be done easily. More advanced terminology constraints, which require runtime terminology browsing or reference subsets retrieval would require more effort but should be manageable once common terminology services for subsets and navigation become more available. The proper handling of EHR Entry subclasses in the target EHR system is probably the most challenging task since some of the Entry types, e.g. INSTRUCTION would need tight integration with applications in order to use the intended semantics correctly.

4. Repeat steps 2 and 3 in iterations until all or most of the archetype semantics are understood and properly implemented in target EHR systems. It is worth noting that the archetype format does not have to be supported 100% before common EHR content models and distributed EHR applications can be established. In other words, most of the existing content models in various proprietary EHR formats would not require the full expressive power of the openEHR archetype format. But it is important to find the subset of the archetype format that can be commonly supported by existing EHR systems in order to start the building of useful applications based on that common ground.

## Discussion

Semantic interoperability between different EHR systems across technical platforms and organizational boundaries requires a consistent way of sharing the syntactic and semantic definitions of clinical content. Archetypes based standards have been developed by the European Standards Committee (CEN), also approved by ISO, for communicating EHR extracts to facilitate the goal of semantic interoperability [15]. In this study we confirm the applicability of the new paradigm using care documentation templates from a large regional EHR installation that has been successfully implemented and running for several years.

A long term goal of using archetype-based systems is to be able to share EHR content between systems and support different reuse scenarios of health data based on standardized archetypes. We believe that even before this long term goal of EHR content exchange can be achieved there are several advantages of having clinical content models expressed as archetypes instead of completely proprietary formats:

• To the EHR system owners (customers), standard-based clinical models mean better protection of their investment in clinical modelling over the years and a lower risk of vendor lock-in.

• Standard-based and freely available archetype tools have better chance than proprietary authoring tools to be used by clinical experts from different healthcare organizations for collaborative authoring and maintenance of the clinical content models across time and space.

• For EHR vendors, this move means less demand on dedicated software for model authoring, thus reduced complexity of software and consequently better focus on the clinical applications instead.

• Clinical content models represented in a common format can speed up the installation and configuration of EHR systems and facilitate the progression of nationally, or even internationally, standardised representation of clinical content, based on which distributed decision support and warnings can be achieved.

• A large repository of legacy archetypes from several vendors and installations could potentially be used as an inspiration and validation when creating a library of "standard" archetypes on regional and national level, which is currently taking place in Sweden. One task when designing archetypes is to try to find the "maximum" dataset for the clinical concept being modelled. A large repository of legacy archetypes is likely to contain some relevant "corner cases" that the modellers have not thought of.

COSMIC templates and openEHR archetypes show a lot of similarity in the way they are used for representing clinical content models. Both models support detailed document structures, validation rules for different data types, terminology bindings to reference terminologies and locally defined terms. However, some of the COSMIC templates from the County of Östergötland have scopes corresponding to several archetypes from the openEHR knowledge repository in a single template and they also seem be tailored for quite specific clinical settings, e.g. outpatient encounter. This makes the conversion of one COSMIC template into a single archetype sometimes inappropriate. It is also noted that within a single COSMIC template, some parts of the template can be substituted by existing archetypes. On the other hand, openEHR templates are introduced to facilitate the use of archetypes through the compositional pattern and further constraints on the included archetypes. From this analysis, some of the COSMIC templates would be better represented as an openEHR template with inclusion of several archetypes instead of just a single archetype. Since the openEHR template object model is directly derived from the archetype object model, it is expected that the representation of existing COSMIC templates as openEHR templates and archetypes would be feasible. Moreover, COSMIC templates that are modelled specifically for some clinical scenarios could be supported by a generic openEHR COMPOSITION archetype with open archetype slots that can be further constrained to satisfy the use case. Different choices between reuse of published archetypes and creation of new archetypes based on parts of the COSMIC template that are to be extracted for later reuse would be interesting to explore in further studies.

Today EHR systems like the Cambio COSMIC that support flexible clinical recording exist [16-18]. The underlying mechanism that enables such flexibility may come with different names, e.g. template support or form designer, but it usually involves the ability of allowing clinical users to choose from a list of common data types, to construct hierarchical structures to group entries and possibly to make links between data entries and terminologies. The expressiveness of these different EHR "template" models varies regarding data types, structural constraints and the ability for handling terminology bindings. Because of wide-spread use of EHR systems based on similar designs in current healthcare enterprises, there exist a great amount of clinical models captured in various proprietary EHR model formats. We believe it is important to preserve many of these precious clinical models; not only are they results of years of continuous refinement based on real clinical experiences, but also for the sake of preserving the meaning of the health records created with these models. Sharing and reuse of these clinical records for different purposes would need access to the original semantic definitions that were used for data capturing. Having a more expressive EHR content model based on international standards is therefore desired. Hopefully this study will serve as a valuable input into further studies into preserving existing clinical models with standards.

This prototype development also shows that the transition from previous generation EHR systems to future generation standards-based systems must not necessarily be a disruptive or an overly resource consuming task. Most, if not all, EHR systems on the market have some template mechanism and the transition can be seen more as an evolution of an existing template mechanism than a total redesign. In this prototype, the information model of Cambio COSMIC was mapped to the reference model of openEHR and the template model of COSMIC was mapped to the archetype model of openEHR. Based on the result of this study, future development of the prototype could include the further alignment of the COSMIC models to the openEHR RM and AM until the translation becomes fully transparent.

The sharing of content models as demonstrated in this paper is an important step in itself but also of course an important step towards the ultimate goal of sharing patient specific clinical data – for direct patient care and for research and quality management that may require the pooling of data from several clinical settings.

### Status

A live software demonstration of converting COSMIC templates to archetypes was successfully performed at the Archetype Workshop at MIE 2008 conference in Gothenburg May 2008 and later at the Danish National SFI Workshop in Copenhagen, November 2008. The conversion prototype software was further enhanced within the context of the Danish archetype trial-out project.

## Conclusion

The result of this study shows that clinical content models from an existing EHR product with a large installed base covering both primary care and specialist hospital care can be consistently represented as archetypes. This finding indicates the applicability of the openEHR archetype methodology and verifies the expressiveness of the openEHR models. It also demonstrated that adding support of the openEHR archetypes to a legacy EHR system could be performed in a smooth and incremental way. Preserving legacy clinical content models using openEHR templates, identifying correct conversion rules for meta-data, and formulating a good strategy to find the correct sub-ENTRY type during conversion will be investigated in future studies.

## Competing interests

The authors declare that they have no competing interests.

## Authors' contributions

RC has contributed to the overall conception and design of the study, developed the conversion rules, implemented the prototype software and drafted the majority of the manuscript. GK has contributed to the initial conception of preserving the legacy clinical content models in standardized format, as well as substantial drafting and critical revising of the manuscript. HÅ has contributed to the initiation of the pilot work on the COSMIC templates from the County of Östergötland, the design idea of the conversion verification protocol and revising of the manuscript. ES and DK have contributed to drafting of the manuscript, and critically revised and commented the manuscript. All authors read and approved the final version to be published.

## Pre-publication history

The pre-publication history for this paper can be accessed here:

http://www.biomedcentral.com/1472-6947/9/33/prepub
